# Two cytosolic glutamine synthetase isoforms play specific roles for seed germination and seed yield structure in *Arabidopsis*

**DOI:** 10.1093/jxb/eru411

**Published:** 2014-10-14

**Authors:** M. Guan, I. S. Møller, J. K. Schjoerring

**Affiliations:** Department of Plant and Environmental Sciences, Faculty of Science, University of Copenhagen, Thorvaldsensvej 40, DK-1871 Frederiksberg C, Denmark

**Keywords:** *Arabidopsis*, cytosolic glutamine synthetase, isoform, mutant, nitrogen, seed germination, seed productivity.

## Abstract

Nitrogen (N) remobilization from reserves to sinks is essential for seedling establishment and seed production. Cytosolic glutamine synthetase (GS1) is up-regulated during both seed germination and seed filling in plants. However, the specific roles of the individual GS1 isogenes with respect to N remobilization, early seedling vigour, and final seed productivity are not known. In this study, impairment of seed germination and seedling establishment is demonstrated in the single knockout mutant *gln1;2*, and the double knockout mutant *gln1;1:gln1;2*. The negative effect of *Gln1;2* deficiency was associated with reduced N remobilization from the cotyledons and could be fully alleviated by exogenous N supply. Following reproductive growth, both the single and double *Gln1;2*-knockout mutants showed decreased seed yield due to fewer siliques, less seeds per silique, and lower dry weight per seed. The *gln1;1* single mutant had normal seed yield structure but primary root development during seed germination was reduced in the presence of external N. *Gln1;2* promoter–green fluorescent protein constructs showed that Gln1;2 localizes to the vascular cells of roots, petals, and stamens. It is concluded that *Gln1;2* plays an important role in N remobilization for both seedling establishment and seed production in *Arabidopsis*.

## Summary

Knockout of glutamine synthetase isogene *Gln1;2* reduces nitrogen remobilization and the number and size of siliques and seeds in *Arabidopsis*. *Gln1;1* affects the response of primary root development to exogenous nitrogen.

## Introduction

Nitrogen (N) is an essential element for plant growth and development (Hawkesford *et al.*, 2012). Plant roots absorb N as nitrate or ammonium (Yuan *et al.*, 2013; Krapp *et al.*, 2014) and these inorganic N forms must be processed before the N can be built into proteins, nucleic acids, and a range of secondary metabolites. A central component in the N processing chain is the enzyme glutamine synthetase (GS; EC 6.3.1.2) which catalyses the assimilation of ammonium into the amide glutamine (Gln) (Hirel *et al.*, 2005). The generated Gln is the main N carrier in plants and can be transported via the xylem and phloem (Rentsch *et al.*, 2007; Zhang *et al.*, 2010).

Besides a key role in primary N assimilation, GS is crucial for reassimilation of NH_4_^+^ which is constantly generated in large quantities in plants via processes such as photorespiration, lignin biosynthesis, and protein turnover (Li *et al.*, 2014). During the latter process, protein-bound N is converted to Gln, promoting recycling of N in storage proteins. This process is critical during seed germination in many plant species, including *Arabidopsis*, when N is remobilized from source organs (cotyledons and hypocotyls) to developing sinks, until the seedling establishes itself as a self-sufficient, autotrophic organism (Fait *et al.*, 2006; Hong *et al.*, 2012). In reproductive growth stages, senescence-induced degradation of leaf proteins provides the main source of N for incorporation into seed storage proteins, and GS activity has been shown to correlate with N remobilization efficiency (Kichey *et al.*, 2005; Guiboileau *et al.*, 2012).

Two GS isoforms exist in higher plants: the cytosolic glutamine synthetase isoform (GS1) encoded by a multigene family, and the chloroplastic glutamine synthetase isoform (GS2) encoded by one gene (*Gln2*) (Swarbreck *et al.*, 2011). In *Arabidopsis*, GS1 is encoded by five individual isogenes with distinct tissue localization and expression patterns as well as distinct affinities for NH_4_^+^ and glutamate (Guo *et al.*, 2004; Ishiyama *et al*., 2004*a*, *b*; Li *et al.*, 2006*a*; Dragicevic *et al.*, 2014). Phylogenetically, the nucleotide and amino acid sequences of these isoforms do not cluster with GS1 sequences from cereals (Thomsen *et al.*, 2014). The function of *Arabidopsis* and cereal GS1 isogenes can thus not be compared directly, highlighting the importance of studying both model and crop species. However, two GS1 isogenes, *Gln1;1* and *Gln1;2*, were up-regulated in *Arabidopsis* lines overexpressing a rice full-length cDNA library under the control of the *Cauliflower mosaic virus* (CaMV) *35S* promoter (Albinsky *et al.*, 2010), indicating a relationship between *AtGS1* isogenes and the rice genes.

Individual GS1 isogenes have been demonstrated to play essential roles in plant development and yield structure in cereal species. This is the case, for example, in maize (*Zea mays*) where *ZmGln1;3* and *ZmGln1;4* are of critical importance for kernel germination (Limami *et al.*, 2002) and for development of the cob with respect to kernel number and kernel size, respectively (Martin *et al.*, 2006; Cañas *et al.*, 2010). In rice (*Oryza sativa*), knockout of *OsGS1;1* resulted in reduced growth and grain filling, while *OsGS1;2* recently was identified as a critical player in primary root NH_4_^+^ assimilation as well as in tillering (Funayama *et al.*, 2013). A crucial function of *OsGS1;1* in coordinating the global metabolic network in rice plants exposed to ammonium as the N source was shown by Kusano *et al.* (2011). In the *OsGS1;1* knockout mutant, the isogenes *OsGS1;2* and *OsGS1;3* were not able to compensate for the loss of *OsGS1;1*, which suggests that these GS1 isogenes are non-redundant (Tabuchi *et al.*, 2005). A recent study in *Brassica napus*, a species closely related to *Arabidopsis*, identified 16 different GS1 isogenes and showed that they were differentially expressed in response to N regimes during leaf senescence (Orsel *et al.*, 2014).

The exact physiological functions of individual GS1 isogenes in relation to plant development and seed productivity are still not known in *Arabidopsis*. The role of individual GS1 isogenes in plant development could partly be related to the quantity of N assimilated or remobilized, and partly to the maintenance of critical N flows and internal N sensing during essential growth stages. It is known that GS1 isogenes in *Arabidopsis* are involved in controlling carbon–nitrogen interactions (Bussell *et al.*, 2013; de Jong *et al.*, 2014) and responses to environmental stimuli such as N form (Engelsberger and Schulze, 2012) and salt stress (Debouba *et al.*, 2013). However, very limited information is available on their specific roles with respect to seedling development and seed yield structure. Lothier *et al.* (2011) examined a *gln1;2* knockout mutant and observed reduced rosette biomass, but only under ample nitrate supply or under provision of ammonium, while the mutant and the wild type (Wt) behaved similarly under nitrate-limiting conditions. In the study of Lothier *et al.* (2011), differences in N remobilization and changes in seed yield structure were only characterized in relative terms, namely as changes in harvest index (the ratio between seed yield and total above-ground biomass) and seed ^15^N partitioning (the ratio between seed ^15^N content and total plant ^15^N content). These parameters did not differ between the *gln1;2* mutant and the Wt, and the same was the case for seed N concentration (Lothier *et al.*, 2011).

The objectives of this work were to test the hypotheses that GS1 isogenes *Gln1;1* and *Gln1;2* play important roles with respect to: (i) N remobilization from reserves in germinating seeds of *Arabidopsis*; (ii) N loading into *Arabidopsis* seeds; and (iii) seed yield structure in *Arabidopsis*. In order to test these hypotheses, three different *Gln1;1* and *Gln1;2* knockout mutants were characterized, namely *gln1;1*, *gln1;2*, and *gln1;1:gln1;2*. It is shown that during germination, *Gln1;2* plays an essential role in seed reserve N remobilization for sink establishment, while *Gln1;1* promotes absorption of exogenous N. In the reproductive growth stage, *Gln1;2* is significant for seed yield structure, affecting the number of siliques, their size, the number of seeds per silique, as well as the dry weight per seed.

## Materials and methods

### Plant material

*Arabidopsis thaliana* ecotype Columbia-0 (Col-0) lines with T-DNA insertions in the 5’-untranslated region, 62 nucleotides upstream of the start codon of *Gln1;1* (*gln1;1*, SALK_000459), and the third intron, at 599 nucleotides downstream of the ATG of *Gln1;2* (*gln1;2*, SALK_145235), were obtained from the European Arabidopsis Stock Centre, Nottingham, UK.

Homozygous *gln1;1* and *gln1;2* mutants were identified by PCR on genomic DNA (Ostergaard and Yanofsky, 2004) with the use of a primer annealing to the left border of the T-DNA (LBa1.3, 5ʹ-ATTTTGCCGATTTCGGAAC-3ʹ) and the following gene- specific primers: for *Gln1;1* (fwd, 5ʹ-TTCACTGTCTTCACCA GGAGC-3ʹ; and rev, 5ʹ-TCCCAAATTTTATTTTAGCATTTAC AG-3ʹ) and for *Gln1;2* (fwd, 5ʹ-CCACAACCACGAACTCTAA AG-3ʹ; and rev, 5ʹ-AACGGAGAATCGAAAAAGAGC-3ʹ). Two PCRs were needed for each plant. One reaction included two gene-specific primers (fwd and rev): the Wt and the heterozygous lines resulted in bands of 1106bp for *Gln1;1* or 1208bp for *Gln1;2*, while the homozygous mutants gave no band. Another reaction was with one gene-specific primer (rev) and one T-DNA primer (LBa1.3): bands were obtained for the homozygous mutants and the heterozygous lines, while the Wt was blank.

*gln1;1:gln1;2* double mutants were generated by crossing the single mutants. F_1_ progeny heterozygous for both genes (*gln1;1*, *Gln1;1/gln1;2*, *Gln1;2*) were identified by PCR using the primers above and were allowed to self-pollinate. The resulting F_2_ progeny (160 plants) were screened by PCR, and seven homozygous mutants, (*gln1;1*, *gln1;1/gln1;2*, *gln1;2*) were obtained. Seeds from self-pollination of these plants were used in the following experiments.

### Plant growth conditions

In soil, single plants were grown in 5.5 litre pots with soil (PINDSTRUP Substrate 2, Pindstrup Mosebrug A/S, Denmark) under controlled growth chamber conditions with an 8 h:16h light:dark cycle, light intensity of 100 μmol m^–2^ s^–1^, 22 °C:20 °C day:night air temperatures, and 75% humidity of the air. N export experiments were performed at 2 weeks after germination (WAG), in order to study N remobilization in early growth stages. Two-week-old *Arabidopsis* seedlings had developed cotyledons and two rosette leaves. The cotyledons were fully expanded and were considered as N sources, and the two rosette leaves were considered to be sinks. Both source and sink organs were harvested in order to compare the N concentration in each part.

At 4 weeks after germination, the plants were transferred to a growth chamber with the same conditions except with a long-day cycle (16 h:8h light:dark) to induce flowering. In order to characterize the reproductive growth of the plants, the numbers of siliques formed on both the main inflorescence stem and the side branches were counted at 8 and 9 WAG. To compare silique development, four flowers per plant with long anthers extending above the stigma were labelled at the pedicel and the corresponding siliques were observed using a stereo fluorescence microscope (Leica MZ FLII) 10 d later. At 10 WAG, fully developed siliques from the main inflorescence stem (bottom 10 siliques) were collected to compare seed number per mature silique. At 12 WAG, desiccated seeds were collected, counted, and weighed.

On Petri plates, seeds were sterilized in 50% ethanol for 1min and in 50% NaClO (Klorin original, Colgate-Palmolive A/S, Denmark) with 0.05% (v/v) Triton X-100 for 10min, then rinsed five times with sterile water to remove the NaClO. Seeds were stratified for 48h at 4 °C in the dark and placed onto modified half-strength Murashige and Skoog (1/2 MS) medium in square Petri plates with three different N treatments. No-nitrogen medium contained 2.5mM KH_2_PO_4_, 2mM MgSO_4_, 2mM CaCl_2_, 0.1mM NaFe-EDTA, 70 μM H_3_BO_3_, 14 μM MnCl_2_, 0.5 μM CuSO_4_, 0.2 μM Na_2_MoO_4_, 10 μM NaCl, 1 μM ZnSO_4_, and 0.3% (w/v) phytagel, pH 5.8. Normal-nitrate medium and high-nitrate medium were similar to the no-nitrogen medium except for the addition of 5mM and 20mM KNO_3_, respectively. The Petri plates were placed quasi-vertically in a growth chamber with an 8 h:16h, light:dark cycle, light intensity of 100 μmol m^–2^ s^–1^, 22 °C:20 °C day:night air temperatures, and 75% humidity of the air. Observations were performed at day 1 and 2 after germination with a stereo fluorescence microscope (Leica MZ FLII). Primary root growth was measured 6 days after sowing (DAS).

### RNA extraction and reverse transcription–PCR (RT–PCR)

Total RNA was extracted from 50–100mg of frozen roots using a solution of 35% (v/v) phenol, 1M guanidine thiocyanate, 1M ammonium thiocyanate, 0.1M sodium acetate, and 5% (v/v) glycerol. Proteins were removed by chloroform, and RNA was purified by isopropanol and washed with ethanol before resuspension in RNase-free water. Following DNA digestion with TURBO DNase (Applied Biosystems/Ambion) and confirmation of RNA quality by gel electrophoresis, reverse transcription was performed using M-MuLV Reverse Transcriptase (New England Biolabs). cDNA was synthesized from 2 μg of RNA estimated from the concentration measured by a nanodrop 2000 spectrophotometer (Thermo Scientific). The resulting cDNAs were used as templates for RT–PCR in order to characterize the mutants at the transcription level. cDNA-specific primers for *Gln1;1* (fwd, 5ʹ-TTCACGTCACCCTCTTCCTC-3ʹ; and rev, 5ʹ-TCATGTCCATTCCAGAACCA-3ʹ) and *Gln1;2* (fwd, 5ʹ-GGTTGGTGGTTCTGGTATGG-3ʹ; and rev, 5ʹ-CTCTCCCGCTGGAGTGTAAG-3ʹ) were synthesized.

### Protein extraction and western blot analysis

Total proteins were extracted from 50mg of frozen shoots using 200 μl of solution [50mM TRIS, 2mM EDTA, 10% (v/v) glycerol, and 10mM 2-merceptoethonal, pH 8.0]. The homogenates were centrifuged at 16 099 *g* for 3min at 4 °C. The supernatant was analysed by western blot analysis. The total protein concentration was determined by a nanodrop 2000 spectrophotometer (Thermo Scientific). A 10 μg aliquot of crude proteins was separated on 12% Criterion XT Bis-Tris gels (Bio-Rad), electrophoretically transferred to 0.2 μm pore-size nitrocellulose membranes (Bio-Rad), and blocked with PBST [8mM K_2_HPO_4_, 3.9mM KH_2_PO_4_, 150mM NaCl, and 0.05% (v/v) Tween-20, pH 7.2] containing 5% skim milk. The blocked membrane was then incubated with 20 μg of anti-GS serum (rabbit IgG) in 20ml of PBST at 4 ºC overnight. After several washes with PBST, the membrane was incubated at room temperature for 2h with horseradish peroxidase-conjugated goat anti-rabbit IgG (1:10 000) (Thermo Scientific), and the immune complexes were detected using chemiluminescence reagents.

### Quantification of N concentration by isotope ratio-mass spectrometry (IR-MS)

The concentration of N was determined in samples of 4–5mg oven-dried finely ground plant material by IR-MS using a system consisting of an ANCA-SL Elemental Analyser coupled to a 20-20 Tracer Mass Spectrometer (SerCon Ltd, Crewe, UK) with acetanilide (Merck) as the standard.

### Cloning of promoter–NLS-GFP-GUS/pCAMBIA 3300 DNA constructs

In order to analyse the expression patterns of *Gln1;1* and *Gln1;2*, the DNA sequences upstream of the genes (promoter sequence) were cloned into a vector from which expression of green fluorescent protein (GFP) will be driven by the respective *Gln1;1* and *Gln1;2* promoters. A 1040bp DNA fragment containing the upstream region of *Gln1;1* and a 1526bp DNA fragment containing the upstream region of *Gln1;2* were commercially synthesized with restriction sites at the 5ʹ and 3ʹ ends of each fragment: G*AGCT*C and C*CCGG*G, for *Sac*I and *Xma*I, respectively. The DNA fragments were cut with *Sac*I and *Xma*I (New England Biolabs), and ligated with the plant expression vector pCAMBIA 3300 NLS-GFP-GUS (Nour-Eldin *et al.*, 2006). PCR amplification of the two DNA fragments with the vector primers fwd, 5ʹ-GAAACAGCTATGACATGATTACGAA-3ʹ; and rev, 5ʹ-AGCAGTTCAACAGCATCATAGGT-3ʹ followed by DNA sequencing confirmed the vectors containing the insertions of *Gln1;1* and *Gln1;2* promoters.

### Plant transformation

Wt *Arabidopsis* Col-0 plants were transformed with *Gln1;1* and *Gln1;2* promoter–NLS-GFP-GUS/pCAMBIA 3300 constructs using the floral dip method (Clough and Bent, 1998). Hundreds of seeds from the transformed plants were sown in soil and transformants were selected by spraying with BASTA. Spraying was performed 1 week after germination and repeated four times at 2 d intervals. At the end of the BASTA selection, transformants continued to grow and remained green, while non-transformed plants were small, turned white, and died. Seeds from self-pollination of the transformants were collected for a further selection of homozygotes.

### Detection of the cell type specific expression of GFP

Observations of green fluorescent nuclei (Chytilova *et al.*, 1999) from *Gln1;1* and *Gln1;2* expression were recorded by confocal microscopy Leica SP5. Roots or floral organs were cut and mounted in water for microscopic observation. A 488nm argon laser was used for the excitation, and the detector was set for emission in the 510–530nm range for GFP, and in the 650–700nm range for the autofluorescence from chloroplasts. Root tissues were from 3-week-old transgenic plants germinated on 1/2 MS medium (Sigma, M5519) containing 1% sucrose solidified with 0.3% (m/v) phytagel, pH 5.8. Floral and reproductive organs were from 6-week-old transgenic plants grown in soil. Similar images were captured from four independent transgenic plants.

## Results

### Characterization of *Arabidopsis* mutants *gln1;1*, *gln1;2*, and *gln1;1:gln1;2*

**Fig. 1.**
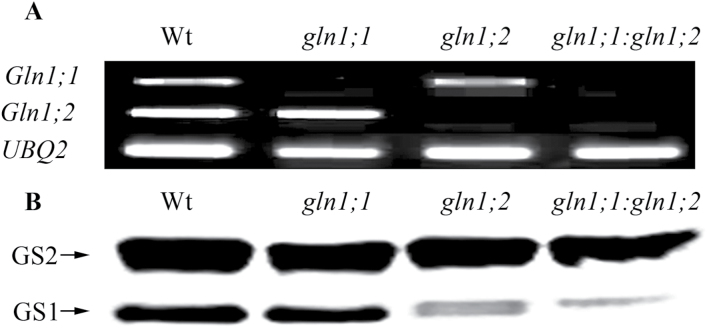
Identification of *Arabidopsis* mutants *gln1;1*, *gln1;2*, and *gln1;1:gln1;2*. (A) RT–PCR analysis of *Gln1;1* and *Gln1;2* expression in the roots of the wild-type (Wt), *gln1;1*, *gln1;2*, and *gln1;1:gln1;2* plants. *UBQ2* was used as a reference gene for equal amounts of cDNA in different samples. Each sample contained a pool of roots from five individual plants. (B) Western blot analysis of GS1 and GS2 contents in shoots of Wt, *gln1;1*, *gln1;2*, and *gln1;1:gln1;2* plants. The upper band corresponds to GS2, and the lower band corresponds to GS1. The same amount of crude protein was loaded in each lane, as described in the Materials and methods. Each sample contained a pool of shoots from five individual plants.

In order to study the effect of a loss of function of *Gln1;1* and *Gln1;2*, homozygous single and double mutants were identified and analysed at the GS gene and protein level. RT–PCR analysis showed that *Gln1;1* was expressed at a much lower level than *Gln1;2* in Wt plants (Fig. 1A). *Gln1;1* expression was absent in *gln1;1* and *gln1;1:gln1;2*, and *Gln1;2* expression was absent in *gln1;2* and *gln1;1:gln1;2* (Fig. 1A). At the protein level, the relative amounts of GS1 and GS2 in crude protein extracted from shoots of *gln1;1*, *gln1;2*, *gln1;1:gln1;2*, and the Wt were estimated by western blot analysis (Fig. 1B). In Wt shoots, two polypeptides were detected. The ~45kDa polypeptide corresponds to GS2, whereas the ~40kDa polypeptide corresponds to GS1. In *gln1;2* and *gln1;1:gln1;2*, a prominent decrease in GS1 protein content was observed, showing that *Gln1;2* is the main shoot isoform in the *Arabidopsis* GS1 multigene family. In *gln1;1*, a similar amount of GS1 protein to that in the Wt was observed (Fig. 1B). This reflects the contribution of protein from other GS1 isoforms, including an up-regulated *Gln1;2* (Guan, 2014).

### Early seedling establishment and N remobilization from cotyledons are impaired in *Gln1;2* knockout mutants

**Fig. 2.**
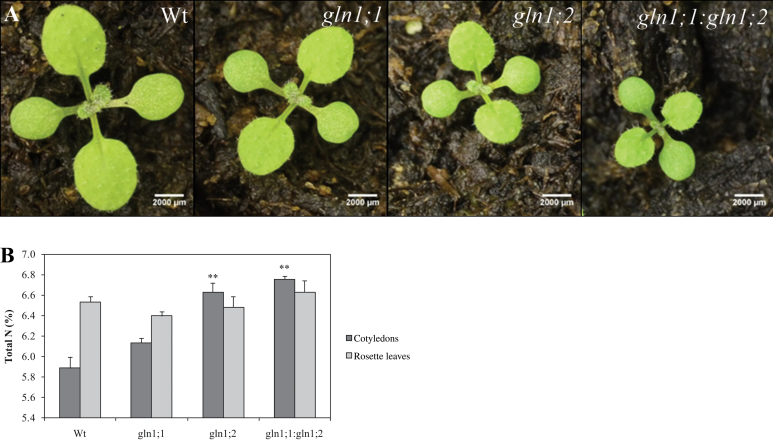
N remobilization in *Arabidopsis* mutants *gln1;1*, *gln1;2*, and *gln1;1:gln1;2* during the first 2 weeks of seedling establishment. (A) Phenotype of the wild-type and mutant seedlings 2 weeks after germination. (B) Total N concentration (% dry weight) in cotyledons (source) and rosette leaves (sink) of the wild type and mutants. Plants were grown in soil for 2 weeks. Results are means ±SE (*n*=4). Asterisks indicate statistically significant differences between the lines, determined using Student’s *t*-test: *P<0.05; **P<0.01. Two independent experiments were carried out with similar results. Scale bars=2000 μm.

Seedling size after 2 weeks of growth in soil was distinctly smaller in the mutants compared with the Wt (Fig. 2A). The size of both the cotyledons and the first real leaf was reduced, most pronouncedly in the *gln1;1:gln1;2* double mutant, followed by the *gln1;2* mutant and the *gln1;1* mutant. The slower seedling development in the mutants suggests a function for *Gln1;2* and, to a lesser extent, *Gln1;1* in N remobilization during seed germination and seedling establishment when N stored in the cotyledons is required to sustain the growth of new sink leaves.

In order to investigate if there was a difference in N remobilization between mutants and the Wt, the N concentration in cotyledons and first rosette leaves was analysed. Plants were grown in soil for 2 weeks, during which period they developed two rosette leaves (Fig. 2A). The Wt plants had a markedly higher N concentration in the new sink leaves than in the source cotyledons (Fig. 2B). The same was the case for the *gln1;1* single mutant, although the difference in N concentration between sink and source leaves was much smaller than in the Wt (Fig. 2B). Both the g*ln1;2* and the *gln1;1*:g*ln1;2* mutants behaved in an opposite manner to the Wt and the *gln1;1* mutant by having a higher N concentration in the source cotyledons compared with the sink leaves (Fig. 2B). This difference was due to the fact that a significantly (*P*=0.01) higher N concentration was maintained in the cotyledons of the two *Gln1;2* knockout mutants *gln1;2* and *gln1;1:gln1;2* relative to the Wt (Fig. 2B), showing that N remobilization from seed reserves in cotyledons was markedly reduced when *Gln1;2* was lacking.

### Seed germination is impaired in the *gln1;2* knockout mutants but the defect can be alleviated by exogenous N

**Fig. 3.**
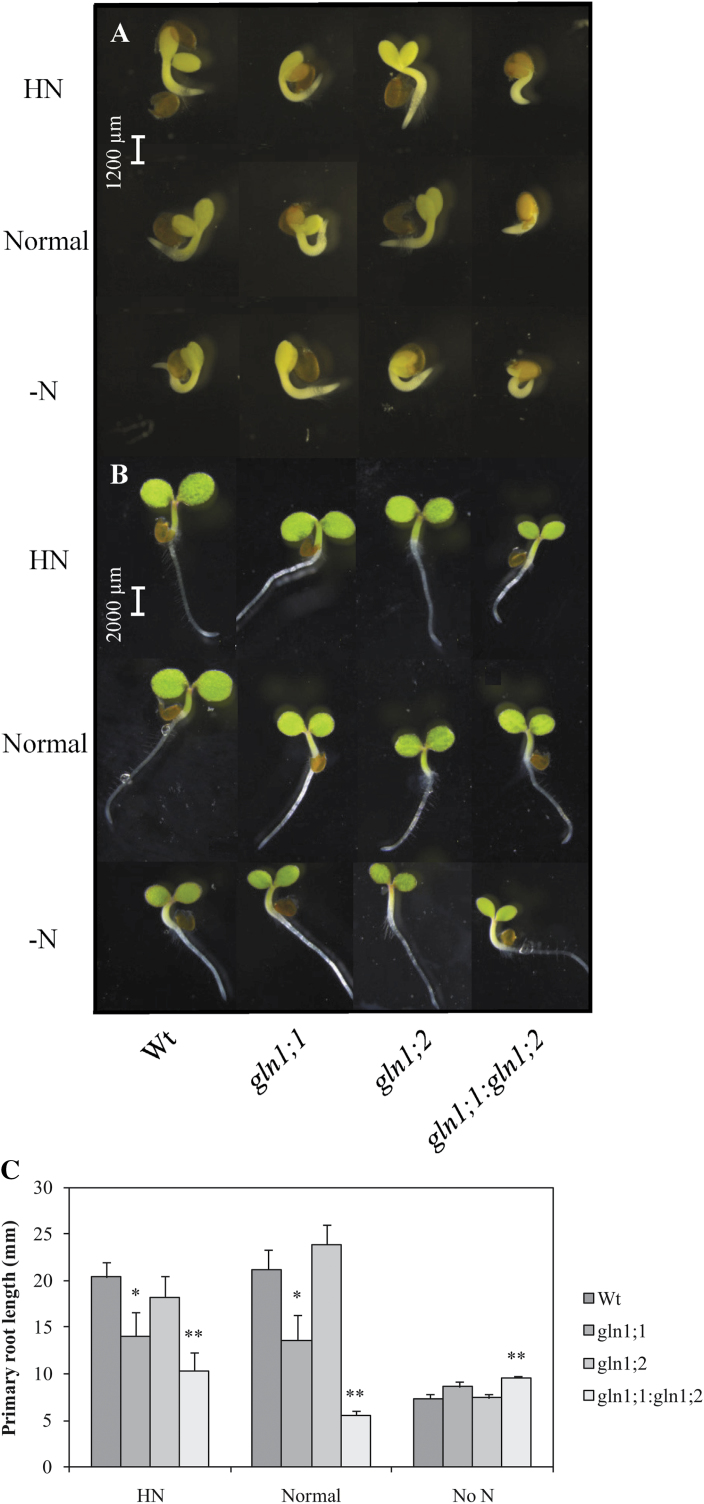
Characterization of *Arabidopsis* mutants *gln1;1*, *gln1;2*, and *gln1;1:gln1;2* in early stages of seed germination. (A) Stereo microscopy images of the wild type and mutants on day 1. (B) Stereo microscopy images of the wild type and mutants on day 2. (C) Primary root length of the wild type and mutants at day 6 (*n*=16–18). The wild-type and mutant seeds were placed onto three different N treatment plates modified from 1/2 MS medium: high-nitrate medium (HN), normal-nitrate medium (Normal), and no-nitrogen medium (–N), as indicated on the left hand side. Plates were placed quasi-vertically. Similar images for (A) and (B) were acquired from four individual seedlings.

In the absence of exogenous N supply (–N), the cotyledons emerging during the first 2 DAS of *gln1;2* and *gln1;1:gln1;2* seeds were breaking through the seed coat more slowly (Fig. 3A) and were distinctly smaller than those from Wt and *gln1;1* seeds (Fig. 3B). In order to study if this phenotype was associated with N deficiency due to reduced N remobilization from seed reserves, an exogenous N supply was applied in the form of nitrate at a normal (5mM) or high (20mM) level to modified 1/2 MS medium.

Addition of N enhanced seed germination (Fig. 3A) and cotyledon size (Fig. 3B) of the *gln1;2* single mutant compared with the –N treatment. At the high level of N supply, the seedling establishment of *gln1;2* was almost fully recovered. In the *gln1;1* mutant, the length of the primary root was negatively affected (*P*=0.05) by exogenous N supply (Fig. 3C), suggesting a function for *Gln1;1* with respect to primary root development in response to external N. The *gln1;1:gln1;2* double mutant had a noticeably retarded germination at all N levels (Fig. 3). Only the radicle was visible at day 1 (Fig. 3A), and the cotyledons were much smaller and had started to turn yellow at day 2 (Fig. 3B). The primary roots developed by *gln1;1:gln1;2* were significantly (*P*=0.01) shorter than in the Wt under high and normal N conditions, but longer in the –N treatment (Fig. 3C).

### Seed yield is significantly reduced in *Gln1;2* knockout mutants

Seed formation requires N remobilization from senescing vegetative plant parts as the N uptake and assimilation during seed filling is insufficient to fulfil the high N demand of the seeds. In order to resolve how knockout of *Gln1;1* or/and *Gln1;2* affects seed production, the seed yield structure in the reproductive growth stage of single and double knockout mutants was characterized.

**Fig. 4.**
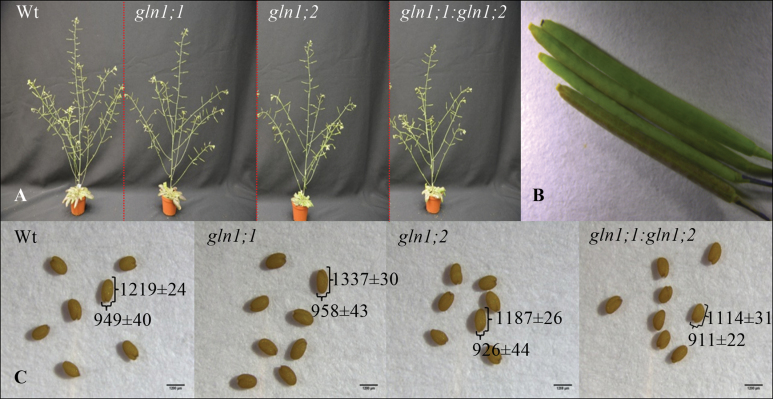
Phenotype of *Arabidopsis* mutants *gln1;1*, *gln1;2*, and *gln1;1:gln1;2* in reproductive growth stages. (A) Images of *gln1;1*, *gln1;2*, *gln1;1:gln1;2* and the wild-type (Wt) plants 8 weeks after germination. (B) Comparison of silique development in the Wt and mutant plants 10 d after flowering. From top to bottom: Wt, *gln1;1*, *gln1;2*, and *gln1;1:gln1;2*. (C) Comparison of length and width of mature seeds harvested from the 10-week-old wild-type and mutant plants. Values (μm) on the vertical and horizontal curly brackets are means ±SE (*n*=10) of seed length and width, respectively. Plants were grown in soil for 4 weeks under short-day conditions, then transferred to long-day conditions in order to induce flowering. Similar images were acquired from four individual plants. Scale bars=1200 μm.

**Fig. 5.**
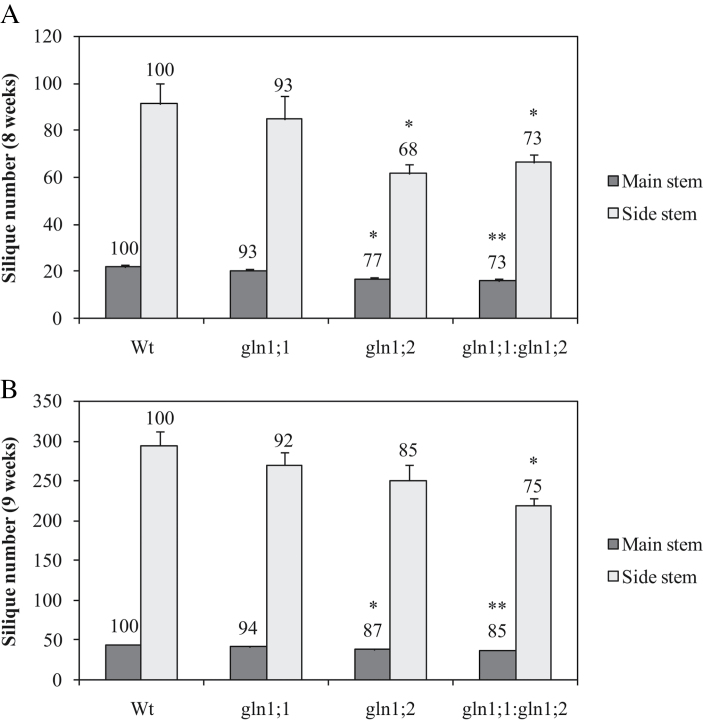
Characterization of siliques from *Arabidopsis* mutants *gln1;1*, *gln1;2*, and *gln1;1:gln1;2*. (A) Number of siliques per plant on the main stem and side branches after 8 weeks of growth. (B) Number of siliques per plant on the main stem and side branches after 9 weeks of growth. Plants were grown in soil for 4 weeks under short-day conditions, then transferred to long-day conditions in order to induce flowering. Results represent means ±SE (*n*=8). Asterisks indicate statistically significant differences between the lines, determined using Student’s *t*-test: *P<0.05; **P<0.01. Values above columns are expressed relative to the wild type set to 100.

Both mutants and the Wt bolted after 7 weeks of growth and developed a main inflorescence stem, with side branches developing after 8 weeks (Fig. 4A). A significantly lower number of siliques was formed in *gln1;2* and *gln1;1:gln1;2* compared with the Wt (Figs 4A, 5A). The decrease in silique number was due to both a lower silique set on the main inflorescence stem (23% reduction for *gln1;2* and 27% for *gln1;1:gln1;2* relative to Wt) and a lower silique set on the side branches (32% and 27% reduction for *gln1;2* and *gln1;1:gln1;2*, respectively; Fig. 5A). One week later, at 9 weeks, a significantly lower number of siliques on both the main inflorescence stem and the side branches was still observed in the *gln1;1:gln1;2* double mutant (Fig. 5B).

**Fig. 6.**
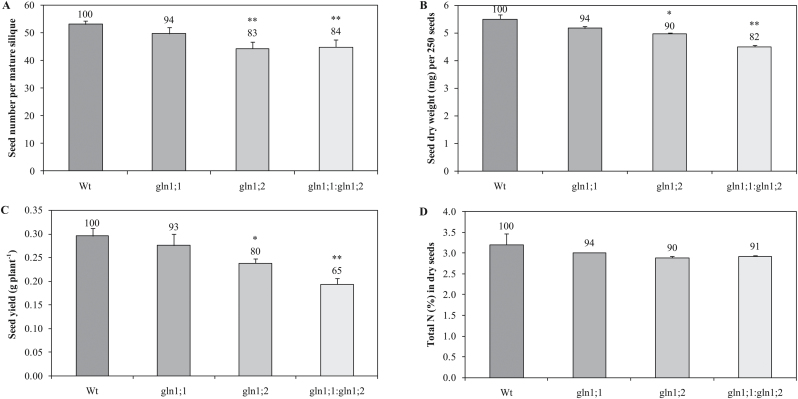
Characterization of mature seeds in *Arabidopsis* mutants *gln1;1*, *gln1;2*, and *gln1;1:gln1;2*. (A) Seed number per mature silique 10 weeks after germination (*n*=10). (B) Dry weight (mg) of 250 seeds harvested 12 weeks after germination (*n*=4). (C) Total seed yield (g) per plant 12 weeks after germination (*n*=8). (D) Total N concentration (% dry weight) in dry seeds 12 weeks after germination (*n*=8). Plants were grown in soil for 4 weeks in short-day conditions, then transferred to long-day conditions in order to induce flowering. Results are means ±SE (*n*≥4). Asterisks indicate statistically significant differences between the lines, determined using Student’s *t*-test: *P<0.05; **P<0.01. Values above columns are expressed relative to the wild type set to 100. Two independent experiments with similar results were carried out.

Shorter siliques were observed in *gln1;2* and *gln1;1:gln1;2* (Fig. 4B), and the size of the mature viable seeds of *gln1;2* and *gln1;1:gln1;2* was smaller relative to that of Wt seeds (Fig. 4C). The seed number per fully developed silique was determined in 10 mature siliques collected from the bottom part of the main inflorescence stem at 10 weeks. Both *gln1;2* and *gln1;1:gln1;2* had an ~17% (*P*=0.01) lower number of seeds per mature silique compared with the Wt (Fig. 6A). In addition, the dry weight of the individual seeds was reduced by 10% in *gln1;2* (*P*=0.05) and by 18% in *gln1;1:gln1;2* (*P*=0.01) (Fig. 6B). The lower seed number and seed dry weight resulted in 20% lower (*P*=0.05) seed yield in the single mutant *gln1;2* and a 35% lower (*P*=0.01) seed yield in the double mutant *gln1;1:gln1;2* (Fig. 6A–C). This clearly shows a crucial role for *Gln1;2* with respect to the seed yield structure in *Arabidopsis*.

### Seed N concentration is unaffected in *Arabidopsis* mutants *gln1;1*, *gln1;2*, and *gln1;1:gln1;2*

The N concentration in the seed dry matter was determined for the mutants and the Wt (Fig. 6D). No significant differences were found between mutants and the Wt with respect to seed N concentration.

### *Gln1;1* and *Gln1;2* are expressed in different cell types in *Arabidopsis*

**Fig. 7.**
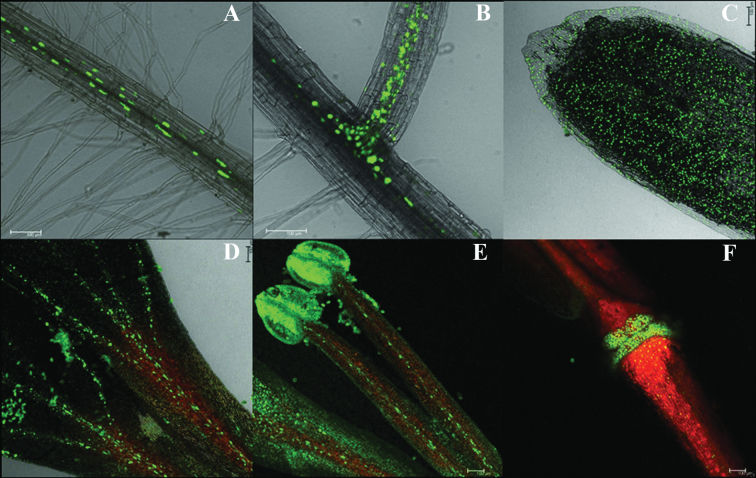
Localization of Gln1;2 in *Arabidopsis* expressing a Pro_*Gln1;2*_–NLS-GFP-GUS promoter–reporter construct. (A) Localization of Gln1;2 in the endodermis of the mature root. (B) Localization of Gln1;2 in the vasculature of the basal region of lateral roots. (C–F) Localization of Gln1;2 in floral and reproductive organs. (C) Sepal. (D) Petal. (E) Stamen. (F) The node between the pedicel and the developing silique. Root tissues for (A) and (B) were from 3-week-old plants germinated on 1/2 MS medium containing 1% sucrose. Floral and reproductive organs for (C–F) were from 6-week-old plants grown in soil. Similar images were acquired from four independent transgenic plants. Scale bars=100 μm.

In order to investigate the localization of *Gln1;2* expression, the Pro_*Gln1;2*_–NLS-GFP-GUS promoter–reporter construct was transformed into *Arabidopsis*. Confocal microscopy of the transgenic roots showed a strong GFP fluorescence in the endodermis of the mature root and in the vasculature of the basal region of lateral roots (Fig. 7A, B). In vegetative shoots, the expression of *Gln1;2* occurred in mesophyll and vascular bundle cells (Guan, 2014). During reproductive growth, bright GFP signals were detected in the epidermal cells of sepals (Fig. 7C). Besides that, fluorescence from *Gln1;2* expression was restricted to cells in the vascular bundle along the veins of petals (Fig. 7D), in the vascular bundle of stamens (Fig. 7E), and in nodes between the pedicel and the developing silique (Fig. 7F). This localization of *Gln1;2* in vascular bundles of floral and reproductive organs agrees with a role for *Gln1;2* in ammonium assimilation for seed production.

The GFP signal driven by the *Gln1;1* promoter was recorded in the epidermal cells of the root elongation zone (Supplementary Fig. S1 available at *JXB* online). This localization supports the observed role of *Gln1;1* in controlling primary root development in interaction with external N availability during seedling early growth (Fig. 3C).

## Discussion

### *Gln1;1* and *Gln1;2* play specific roles in seed germination and seedling establishment in *Arabidopsis*

In *Arabidopsis*, >90% of seed N is incorporated into storage proteins (Baud *et al.*, 2002; Li *et al.*, 2006*b*). When these storage proteins are degraded during germination (Hong *et al.*, 2012), ammonium is produced and needs to be reassimilated into Gln for subsequent remobilization to support seedling growth (Rentsch *et al.*, 2007). However, the specific roles of the individual GS1 isogenes with respect to N remobilization from seed storage proteins during germination have not been clarified. In the present study, it is reported that lack of *Gln1;2* implies negative consequences for both seed germination (Fig. 3) and early seedling establishment (Fig. 2). Although the defect in seed germination of the *gln1;2* mutant could be almost fully recovered by exogenous N supply (Fig. 3), early seedling establishment after 2 weeks of germination in soil with access to external N was nevertheless markedly reduced (Fig. 2). This illustrates that germination and remobilization of seed reserves are regulated independently (Pritchard *et al.*, 2002; Fait *et al.*, 2006) and, although primary roots are capable of absorbing external N during germination (Kircher and Schopfer, 2012), the transition between the use of seed and external resources may not be able to fully restore early growth. As shown here, *Gln1;2* plays an essential role with respect to early seedling establishment by promoting N remobilization from seed reserves, and loss of this function cannot be fully compensated for by absorption of N from the surroundings.

In order to support seedling establishment, N absorbed from the soil must be assimilated into Gln in the roots, then transported to the shoots (Rentsch *et al.*, 2007; Zhang *et al.*, 2010). The GFP signal driven by the *Gln1;1* promoter was recorded in the epidermal cells of the root elongation zone (Supplementary Fig. S1 at *JXB* online; see also Ishiyama *et al.*, 2004*b*). This localization suggests that *Gln1;1* plays a role with respect to assimilation and sensing of external N. Root architecture is modulated in response to external N availability (Giehl *et al.*, 2014; Ma *et al.*, 2014) and also the provision of ammonium or nitrate may trigger lateral root branching and reduce the length of the primary root axis (Lima *et al.*, 2010; Yan *et al.*, 2014). The shorter primary roots developed by the *gln1;1* mutant in response to application of external N (Fig. 3) suggest that *Gln1;1* constitutes part of the signalling pathway mediating modifications of the root system in response to external N supply.

In the *gln1;1:gln1;2* double mutant, a cumulative effect by knockout of both *Gln1;1* and *Gln1;2* was observed, which severely retarded both seed germination and seedling establishment (Figs 2, 3). The stunted phenotype reflects both impaired utilization of external N due to lack of *Gln1;1* and decreased N remobilization from cotyledons due to lack of *Gln1;2*. When exposed to external N, the double mutant had 40–80% shorter primary roots than the Wt and the *gln1;2* mutant (Fig. 3C). In contrast, in the absence of external N, the double mutant had the longest roots among the lines (Fig. 3C), suggesting a more pronounced N deficiency causing a relative stimulation of root extension (Fischer *et al.*, 2013).

### *Gln1;2* plays an important role in seed yield structure

Uptake and assimilation of N during vegetative growth stages are critical processes for generation of the biomass infrastructure and phytohormonal signals which are required for initiation and maintenance of inflorescences and seed meristems (de Jong *et al.*, 2014). Later on during reproductive growth stages, N in senescing leaves must be remobilized to ensure N for proper growth of the developing seeds (Diaz *et al.*, 2008; Guiboileau *et al.*, 2013). For efficient N remobilization to seeds, Gln is loaded into the phloem of the source organs and transported to the reproductive sinks (Taylor *et al.*, 2010; Zhang *et al.*, 2010). Direct evidence for specific roles for individual GS1 isogenes in seed yield structure and N remobilization in *Arabidopsis* has so far not been obtained.

Total seed yield in the *gln1;2* single mutant and in the *gln1;1:gln1;2* double mutant was significantly reduced compared with the Wt. The reduction in seed yield was due to a lower number of siliques per plant (Figs 4A, 5), a lower number of seeds per silique (Figs 4B, 6A), and lower weight per seed (Figs 4C, 6B). Thus, all three components of the seed yield structure were negatively affected by the knockout of *Gln1;2*, which is the main GS1 isoform in the shoot (Fig. 1B). Lothier *et al.* (2011) studied a different *Atgln1;2* mutant and concluded that *Gln1;2* was not essential for seed production or internal N distribution between vegetative and reproductive shoot components. However, the study by Lothier *et al.* (2011) was limited to a measurement of relative parameters only, such as the harvest index (seed weight as a proportion of total shoot weight) and the seed ^15^N partitioning (the ratio between seed ^15^N content and total plant ^15^N content). Ratios between yield parameters can be similar even though the absolute values are markedly different (Zheng *et al.*, 2013). This was also the case in the present work where the proportion of ^15^N in different organs of *gln1:2* and Wt plants at maturity was similar despite a significantly lower absolute quantity of ^15^N in the different plant parts of the mutant compared with the Wt (Supplementary Fig. S2 at *JXB* online). Much more N was thus handled by the Wt plants, reflecting the larger size of these plants (Fig. 4), but in relative terms *gln1;2* did not affect the pattern of N distribution. This suggests that *Gln1;2*, as well as having an effect on the absolute quantity of N remobilized, also plays a role in establishment of the actual yield capacity by maintaining sufficient N flows during critical growth stages. In maize, *ZmGln1;3* and *ZmGln1;4* were shown to play specific roles in the development of the cob with respect to kernel number and kernel size, respectively (Martin *et al.*, 2006; Cañas *et al.*, 2010) suggesting a similar role for GS1 isogenes with respect to internal N signalling.

While seed size and weight were reduced in the *Gln1;2*-knockout mutants (Fig. 6B), seed N concentration was not affected (Fig. 6D). During seed filling, the rate of increase in seed weight following import of photoassimilates is normally higher than the rate of increase in seed N accumulation (Corby *et al.*, 2011). However, in the *Gln1;2* knockout mutants, the smaller seed size reduced the volume to be filled with N during seed growth, thus allowing the N concentration in the individual seeds to be sustained.

Using promoter–GFP constructs, a clear tissue- and cell type-specific localization of *Gln1;2* expression was observed not only in the root vasculature and in the leaf veins (Fig. 7; Ishiyama *et al.*, 2004*b*; Lothier *et al.*, 2011), but also in the epidermal cells of sepals, in the veins of petals and stamens, and in nodes between the pedicel and the developing silique (Fig. 7). This localization ties in with a role for *Gln1;2* in N translocation and redistribution via the xylem and phloem, respectively (Martin *et al.*, 2006; Bernard *et al.*, 2008). In conjunction with, for example, NH_4_^+^ transporters in the stamen (Yuan *et al.*, 2009), *Gln1;2* may further specifically catalyse N transport to reproductive sinks such as pollen and siliques, thereby constituting a causal link with seed yield structure.

## Conclusions

The glutamine synthetase isogene G*ln1;2* plays an important role in N remobilization from reserves in germinating seeds and in development of seed yield components in *Arabidopsis*. The isogene G*ln1;1* affects the response of primary root development to exogenous N provision during seed germination in *Arabidopsis*.

## Supplementary data

Supplementary data are available at *JXB* online.

Figure S1. Localization of *Gln1;1* in epidermis cells in the elongation zone of 3-week-old *Arabidopsis* roots.

Figure S2. ^15^N content in different tissues of *gln1;2* and *Arabidopsis* wild type.

Supplementary Data
