# Supplementary material for: Two cytosolic glutamine synthetase isoforms play specific roles for seed germination and seed yield structure in Arabidopsis
Source: J Exp Bot. 2014 Oct 14;66(1):203–12. doi: 10.1093/jxb/eru411 (PMC4265158; doi:10.1093/jxb/eru411)
Supplement: Supplementary Data [file supp_66_1_203__index.html]

Two cytosolic glutamine synthetase isoforms play specific roles for seed germination and seed yield structure in Arabidopsis — Two cytosolic glutamine synthetase isoforms play specific roles for seed germination and seed yield structure in Arabidopsis — Supplementary Data 

# Two cytosolic glutamine synthetase isoforms play specific roles for seed germination and seed yield structure in *Arabidopsis*

## Supplementary Data

Data files

**Files in this Data Supplement:**

- Supplementary Data - Supplementary Data
