# Supplementary material for: Two cytosolic glutamine synthetase isoforms play specific roles for seed germination and seed yield structure in Arabidopsis
Source: J Exp Bot. 2014 Oct 14;66(1):203–12. doi: 10.1093/jxb/eru411 (PMC4265158; doi:10.1093/jxb/eru411)
Supplement: Supplementary Data [file supp_eru411_jexbot131805_file001.pdf]

Supporting Information

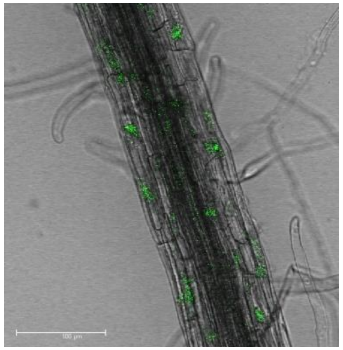

Figure S1. Localization of Gln1;1 in epidermis cells in the elongation zone of 3-week-old *Arabidopsis* roots. Bars = 100 µm.

Supporting Information

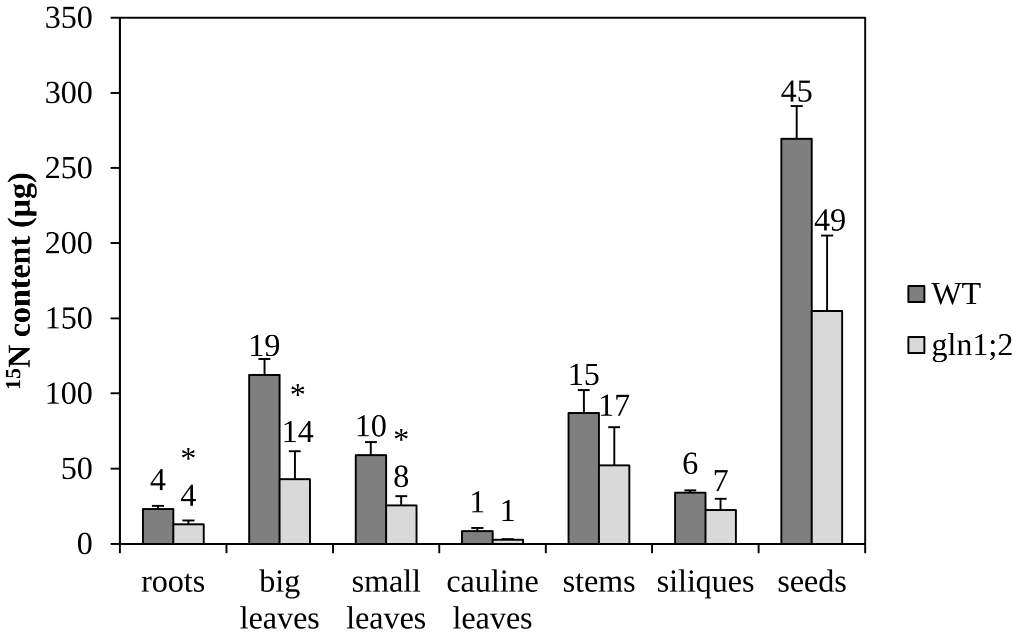

Figure S2. ^{15}N content in different tissues of *gln1;2* and *Arabidopsis* Wt.
